# Dynamics and interactions of ibuprofen in cyclodextrin nanosponges by solid-state NMR spectroscopy

**DOI:** 10.3762/bjoc.13.21

**Published:** 2017-01-27

**Authors:** Monica Ferro, Franca Castiglione, Nadia Pastori, Carlo Punta, Lucio Melone, Walter Panzeri, Barbara Rossi, Francesco Trotta, Andrea Mele

**Affiliations:** 1 Department of Chemistry, Materials and Chemical Engineering “G. Natta”, Politecnico di Milano, Piazza L. da Vinci 32 – 20133 Milano, Italy; 2 Università degli Studi e-Campus, Via Isimbardi 10, 22060 Novedrate, Como, Italy; 3 CNR-ICRM, Via L. Mancinelli 7, 20131 Milano, Italy; 4 Elettra – Sincrotrone Trieste, Strada Statale 14 km 163.5, Area Science Park, 34149 Trieste, Italy; 5 Department of Physics, University of Trento, via Sommarive 14, 38123 Povo, Trento, Italy; 6 Department of Chemistry, University of Torino, Via Pietro Giuria 7, 10125 Torino, Italy

**Keywords:** cross-polarization, cyclodextrin, ibuprofen, nanosponges, solid-state NMR

## Abstract

Two different formulations of cyclodextrin nanosponges (CDNS), obtained by polycondensation of β-cyclodextrin with ethylenediaminetetraacetic acid dianhydride (EDTAn), were treated with aqueous solutions of ibuprofen sodium salt (IbuNa) affording hydrogels that, after lyophilisation, gave two solid CDNS-drug formulations. ^1^H fast MAS NMR and ^13^C CP-MAS NMR spectra showed that IbuNa was converted in situ into its acidic and dimeric form (IbuH) after freeze-drying. ^13^C CP-MAS NMR spectra also indicated that the structure of the nanosponge did not undergo changes upon drug loading compared to the unloaded system. However, the ^13^C NMR spectra collected under variable contact time cross-polarization (VCT-CP) conditions showed that the polymeric scaffold CDNS changed significantly its dynamic regime on passing from the empty CDNS to the drug-loaded CDNS, thus showing that the drug encapsulation can be seen as the formation of a real supramolecular aggregate rather than a conglomerate of two solid components. Finally, the structural features obtained from the different solid-state NMR approaches reported matched the information from powder X-ray diffraction profiles.

## Introduction

In the last ten years cyclodextrin nanosponges (CDNS) polymer materials received great attention as promising new materials in several fields of applications such as bio-catalysis, agriculture, analytical chemistry [1–2] and in pharmaceutical research. Their wide applicability is mostly due to their nanoporous structure, along with their high chemical and thermal stabilities. Considering the pharmaceutical applications, they received interest as drug carriers, since these materials are able to accommodate small drug molecules within their porous network made of CD lipophilic cavities and more hydrophilic polymer channels originated during the cross-linking process with suitable cross-linking agents (CL). The polymer is obtained by condensation reaction of the OH groups of the glucopyranose units of cyclodextrins (CD) with a poly-functional cross-linking agent [3]. CDNS have been characterized, in the solid state, by a repertoire of physical methods such as solid-state ^13^C CP-MAS NMR, FTIR and Raman spectroscopy [4–6]. Moreover, in many of their formulations, CDNS showed good swelling capability when contacted with water solutions, giving rise to homogeneous hydrogels potentially useful as drug carriers [7]. In order to obtain an effective control over the drug delivery procedure, several efforts have been made to explore how the nanoporous polymer structure influences the delivery property of entrapped drugs [8]. In particular the transport properties of ibuprofen sodium salt (IbuNa) entrapped in CDNS polymer gels has been investigated by high resolution magic angle spinning (HR-MAS) NMR technique [9] as a paradigmatic case of an active pharmaceutical ingredient (API) entrapped in a polymeric scaffold. The results pointed out that the motion of a small drug molecule drastically changes from subdiffusive to slightly superdiffusive regimes depending on the cyclodextrin to CL molar ratio, and hence on the CDNS polymeric structure. Ibuprofen is the API of many nonsteroidal anti-inflammatory formulations widely used in the treatment of fever, rheumatoid arthritis and other inflammatory diseases [10]. From the chemical viewpoint, ibuprofen is the (*RS*)-2-(4-(2-methylpropyl)phenyl)propanoic acid. Its sodium salt is water-soluble and absorbed in blood plasma more quickly than the undissociated acid [11]. In the following, we will refer to the undissociated acid as IbuH and to the sodium salt as IbuNa. For both IbuH and IbuNa, the desired pharmacological effects are due to the *S*-enantiomer. Nevertheless, the commercially available drug is the racemic mixture (*R,S*)-ibuprofen. Moreover, solid-state NMR spectra revealed that in all tablet samples, ibuprofen is present in acidic form IbuH with different contents of bound water within tablets [12] depending on the formulation.

In this work the racemic (*R,S*)-ibuprofen sodium salt (IbuNa) was encapsulated in cyclodextrin nanosponges (CDNS) obtained by cross-linking of β-cyclodextrin with ethylenediaminetetraacetic acid dianhydride (EDTAn) in two different preparations: CDNS(1:4) and CDNS(1:8), where the 1:*n* notation indicates the CD to EDTAn molar ratio used for the synthesis. The solid state products were prepared via freeze-drying of the hydrogels obtained by swelling the nanosponge with aqueous solutions of IbuNa. The main purpose of the present work is to investigate the structural changes of the host CDNS material as well as the drug chemical and structural modifications in the polymer network in the solid state. The methodological approach here presented relies upon two different solid state NMR methodologies and it is finally supported by powder X-ray diffraction (PXRD) data. In particular, here we present the use of variable contact time (VCT) cross-polarization ^13^C NMR as a novel and powerful source of information on the drug loaded polymers, complementary to the chemical shift data achievable with the commonly employed ^13^C CP-MAS NMR spectra. In a typical VCT experimental session, an array of CP-MAS spectra are collected by modulating the contact time needed for cross polarization. The experimental data can be fitted by using suitable theoretical models. When feasible, the fitting procedure affords important relaxation parameters such as the proton spin-lattice relaxation times *T*_1ρ_(H) and the ^1^H–^13^C cross-polarization time constant *T*_CH_. In general, the outcome of VCT data processing is a “dynamic fingerprint” that can be used for the characterization of the polymer-drug system.

In general, the CP-VCT methodology can be exploited to provide a dynamic characterization of polymeric systems which are expected to be structurally similar, as recently demonstrated for molecular imprinted epichlorohydrin–cyclodextrin polymers [13]. In the present work, CDNS(1:4) and CDNS(1:8), along with the corresponding ibuprofen loaded systems, are used as a paradigmatic case. The CDNS do not show significant chemical shift variations on passing from the unloaded polymers to the drug-loaded systems, thus making their characterization difficult. The dynamic fingerprint provided by the VCT data allows overcoming this problem. In a broader sense, CP-VCT techniques can be considered a convenient approach for systems in which the common NMR parameters, like chemical shift and line-width, may fail to provide an acceptable characterization due to high structural similarity at molecular level.

Finally yet importantly, in the present work we also report on solid state ^1^H NMR spectra of the examined compounds acquired under fast magic angle spinning (Fast MAS) conditions. As detailed in the Discussion, this technique is quite rarely used. The results of the ^1^H Fast MAS NMR experiments provided further structural details of the state of the drug molecule loaded in the CDNS materials.

## Theoretical Aspects

### The CP-MAS experiment and CP dynamics

Cross-polarization (CP) is a SSNMR technique originally developed for enhancing the signal intensities of low-abundance spins, generally referred to as *S* spins (typically ^13^C, ^15^N, ^29^Si etc.) by polarization transfer from high-abundance spins, in turn labelled as *I* spins (usually protons). The transfer of magnetization occurs via heteronuclear dipolar interaction between the two spin species (*I–S*) when the Hartmann–Hahn condition is met. In the present work only ^1^H,^13^C magnetization transfer will be considered, thus the Hartmann–Hahn condition can be expressed as: γ(^1^H)B_1_(^1^H) = γ(^13^C)B_1_(^13^C), where γ are the gyromagnetic ratios of the nuclei and B_1_ is the so-called spin-lock field generated by the spectrometer acquisition hardware. The efficiency of the CP process strongly depends on the structural and dynamic properties of the system, in particular molecular conformational changes, rotational dynamics and internuclear distances in the solid state. The CP dynamic regime can be explored by acquiring several experiments at increasing contact time (CT). This methodology is called Variable Contact Time (VCT); generally, the CT is increased from few μs to some ms. A quantitative analysis of the CP data can be performed by fitting the experimental curves of the CP intensity, *I*(t), versus the contact time *t*. The fitting procedure follows several theoretical models. A complete description of the theory and practical considerations is reported in the review article of Kolodziejski and Klinowski [14]. In the following, we will describe in detail two models: the classical *I–S* model and the I–I*–S model.

#### The classical *I–S* model

This theoretical model, initially developed by Mehring [15], is based on the classical spin thermodynamics. The system is described as consisting of a lattice with a huge heat capacity and two subsystems, the isolated *S* spins and an extended network of coupled *I* spins. The heat capacity of the rare *S* spins is much lower than that of *I* spins. According to this model, the spins *I* are characterized by a rapid spin diffusion so that they behave as a single spin system at a uniform spin temperature.

**Figure 1:**
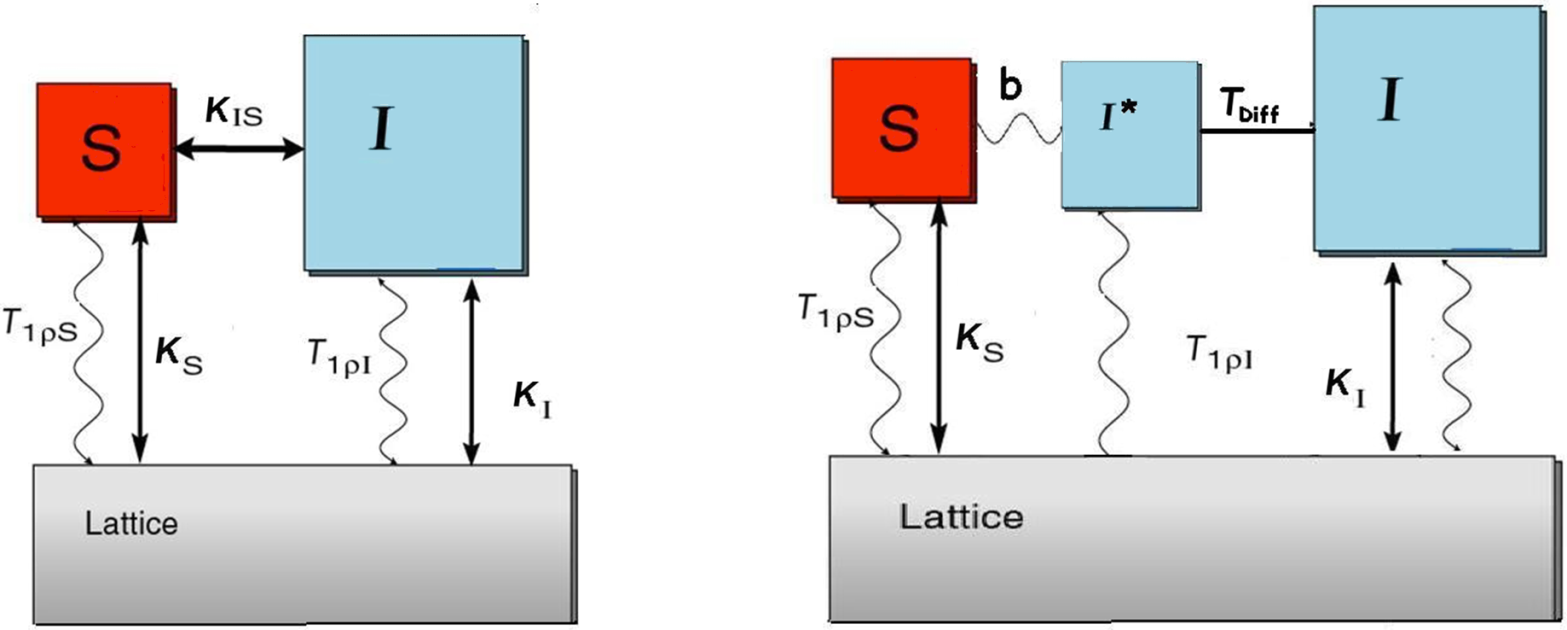
Schematic thermodynamic description of the CP process and time constants. The *I-S* model (left), the I–I*–S model (right). Adapted with permission from [14]. Copyright 2002 American Chemical Society.

After the initial excitation pulse of the *I* spins, when the Hartmann–Hahn condition is met the magnetization is transferred from the *I* spins to the observed *S* spins. The schematic representation of the CP process is shown in the left-hand part of Figure 1.

The enhanced signal of the *S* spins detected as a function of the of cross-polarization contact time, *t*, is expressed as follows (Equation 1):

[1]
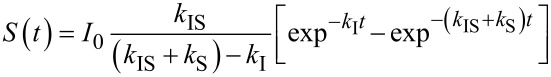


where *k*_IS_ is the rate constant for the “heat flow” between the I and S spins, and *k*_I_, *k*_S_ are the inverse of the spin-lattice relaxation times in the rotating frame for the spin *I* (*T*_1ρ_(H)) and *S* (*T*_1ρ_(S)), respectively. The simplified Equation (2) is obtained assuming that: 1) *k*_S_ < *k*_IS_ (*k*_S_ is negligible), 2) *k*_IS_ + *k*_S_ > *k*_I_, the system is in the fast CP regime.

[2]
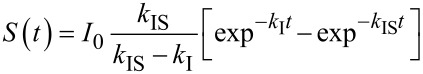


According to Equation 2, the magnetization of the *S* spins rises with the CP rate constant *k*_IS_, reaches the maximum *S*_CPMAX_ at time *t*_MAX_ and, subsequently, decreases with the time constant *k*_I_. Some experimental curves showing the trends mentioned before are shown in Figure 7. Finally, the two relaxation parameters *T*_CH_ and *T*_1ρ_(H) can be determined, in principle, by fitting the experimental curves with the bi-exponential Equation 2.

#### The I–I*–*S* model

In the I–I*–S model, the reservoir of the *I* (^1^H) nuclei surrounding a given *S* (^13^C) nucleus is divided into two subsets: I* indicates the protons closest to the observed S nucleus, *I* the nuclei at longer distances. This model was proposed to explain the case when the proton spin diffusion rate 1/*T*_Diff_ is not fast enough so that the *I* spins do not behave as a whole spin system. The I*–*S* spin pairs are isolated from the spin network and exchange polarization in an oscillatory mode. The oscillations are damped by the spin-diffusion contact with the whole *I* spin system. A schematic representation of the physical process is reported in the right-hand side panel of Figure 1. The oscillatory CP kinetics is described [16] by the Equation 3:

[3]
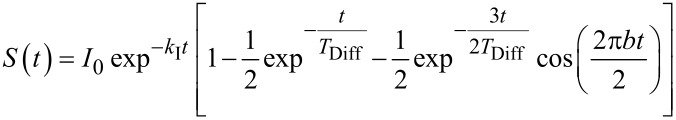


where *b* is the heteronuclear dipolar coupling. Further mathematical developments allow determining the composition of the spin cluster.

The oscillatory behaviour of the signal intensity in ^1^H–^13^C VCT experiments was observed for the first time on single crystals [16–18]. It was found that the frequency of oscillation depends on the heteronuclear dipolar coupling *b* due to the orientation of the single crystal respect to the external magnetic field. Later on, similar results have been found for other systems, including powders, bilayers, and supramolecular complexes [19].

Comparing the two models, it was demonstrated that the I–I*–S model applies in special cases when the whole spin system has strong I*–*S* dipolar interactions and weak homonuclear *I*-I* dipolar couplings [14]. In the opposite case, i.e., when weak I*−*S* and strong *I*−I* couplings are present, the CP kinetics follow the more general *I*−*S* model. As a final remark, we wish to stress that the oscillatory behaviour described above has not been reported frequently so far, and that the data reported and discussed in the following of this work represent an example.

## Results and Discussion

### Solid-state NMR

#### ^1^H MAS NMR spectroscopy

^1^H high-resolution spectra of small organic molecules, characterised by the isotropic chemical shift and *J* couplings, are easily obtained in solution. By contrast, for rigid solids, the corresponding solid-state ^1^H NMR spectrum generally shows broad, featureless lines due to the strong homonuclear dipolar couplings among protons, which in many cases exceed the range of chemical shifts. Consequently, solid-state ^1^H NMR spectra are seldom reported, the assignment of the ^1^H resonances and quantitative analysis of *SS* ^1^H NMR spectra is still very challenging. The spectral resolution can be improved by applying line narrowing techniques such as magic angle sample spinning (MAS) [20], usually combined with complex homonuclear decoupling pulse sequences designed ad hoc [21–22]. The resolution of the solid-state NMR signals strongly depends on the sample spinning speed, for diluted nuclei (^13^C or ^15^N) slow or moderate speeds are enough to obtain resolved spectra, while fast or very fast spinning (spinning regime >50 kHz) conditions are needed to obtain “liquid like spectra”.

**Figure 2:**
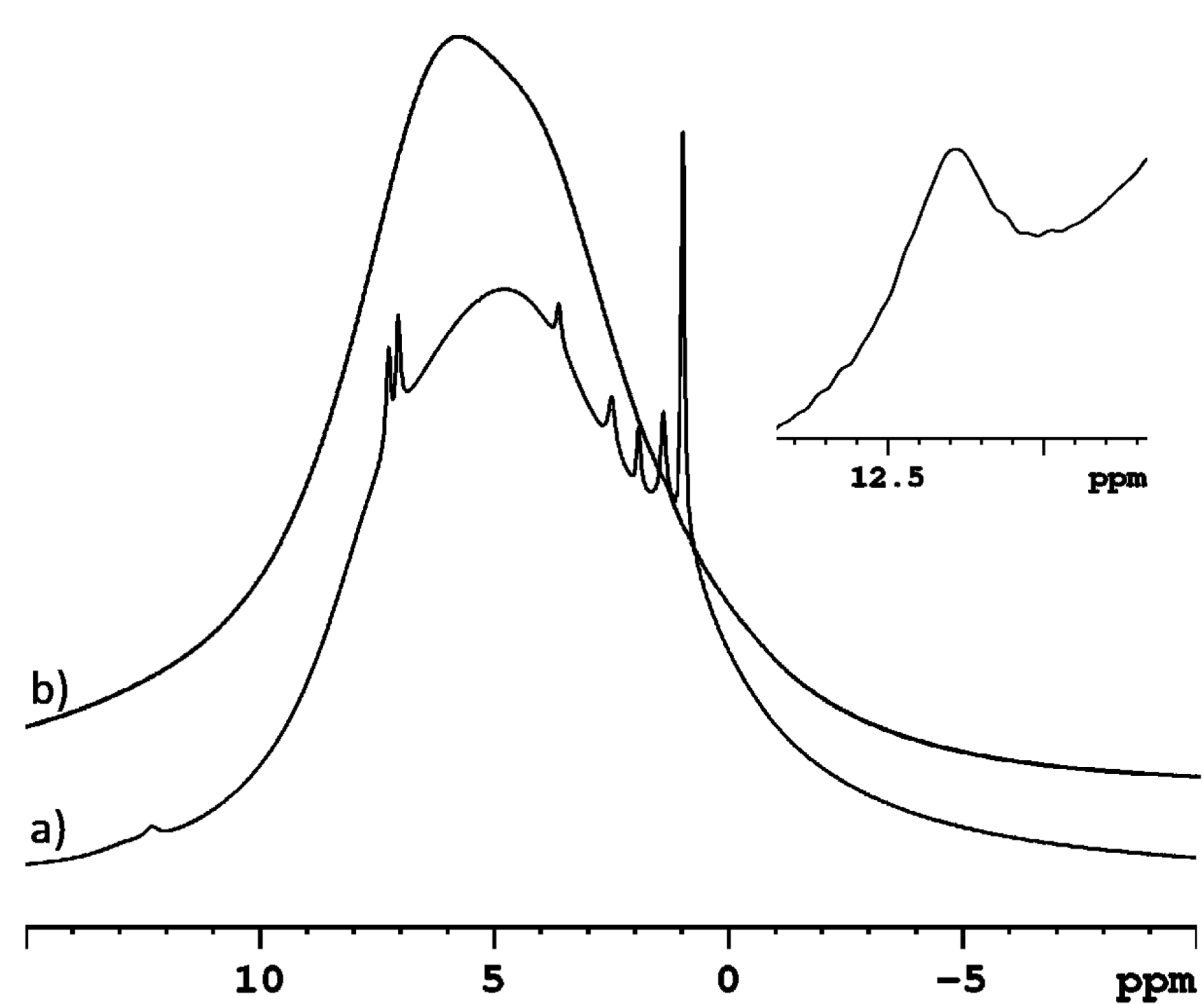
^1^H MAS NMR spectra of a) CDNS(1:8)-IbuNa and b) CDNS(1:8). The inset (top right) shows the expansion of the spectral region where the hydrogen bonded protons of the carboxylic functional groups resonate. The notation CDNS-IbuNa indicates the original preparation, irrespective of the conversion of the sodium salt into the undissociated acid.

We begin the spectroscopic investigation of the systems by discussing the proton MAS spectra of the two nanosponges CDNS(1:8) and CDNS(1:4) free and IbuNa loaded according to the procedure described in the experimental section. The ^1^H MAS NMR spectra of both nanosponges CDNS(1:8) and CDNS(1:4) (Figure 2b), acquired at 20 kHz spinning speed, exhibit broad lines characteristic of a rigid solid due to a not complete averaging of the strong homonuclear ^1^H–^1^H dipolar interactions. A different NMR pattern is observed for the CDNS-drug samples. The ^1^H spectrum of the sample CDNS(1:8)-IbuNa (Figure 2a) shows narrow resolved signals of the drug molecule and a broad line for the polymeric network. Similarly, the ^1^H MAS spectrum (Figure 2b) of CDNS(1:4)-IbuNa shows resolved peaks (line widths of 40–50 Hz) of the drug molecule.

**Figure 3:**
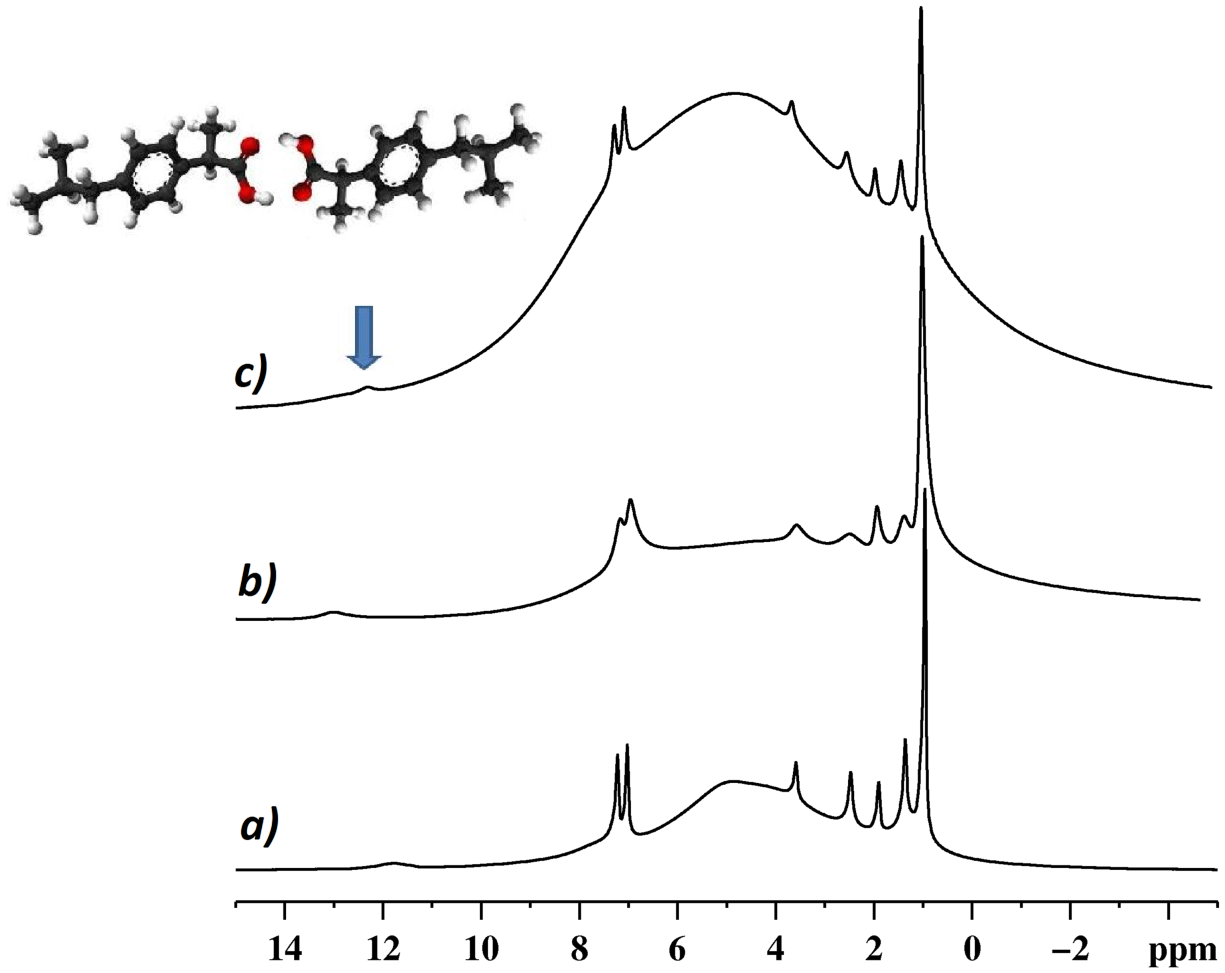
The effect of the spinning speed on the spectral features. ^1^H MAS NMR spectra of a) CDNS(1:4)-IbuNa under conditions of fast spinning speed (40 kHz), b) CDNS(1:4)-IbuNa recorded at lower spinning speed (20 kHz), c) CDNS(1:8)-IbuNa recorded at 20 kHz spinning speed. The notation CDNS-IbuNa indicates the original preparation, irrespective of the conversion of the sodium salt into the undissociated acid. The arrow indicates the peak assigned to the hydrogen bonded dimer, sketched in the inset.

Signal resolution further increases under fast MAS (40 kHz spinning) conditions (Figure 3c). The observed high resolution of the IbuNa spectral lines is the consequence of the efficient averaging of the homonuclear ^1^H–^1^H dipolar interactions due to fast rotation of the sample at the magic angle. All the resonances can be easily assigned. The peak at 0.96 ppm is due to the methyl groups, small peaks belonging to the alkyl protons are in the range 1.4–3.6 and the resonances at 7–7.23 are due to the phenyl group. It is important to observe a high frequency signal at 12.05 ppm for CDNS(1:8) and at 13 ppm for CDNS(1:4). This peak is indeed unexpected, as it is assignable to carboxylic protons involved in hydrogen bonds. The presence of such a signal provides evidence for two remarkable facts: i) the drug entrapped in the nanosponge is in the acidic form, and ii) the ibuprofen molecules hosted in the CDNS are fully involved in a hydrogen-bond network. This point is worth of a comment: racemic IbuH was reported [23] to form dimers in the crystal state, as assessed by the combined single crystal X-ray and pulsed neutron diffraction techniques. In the present study, the observed ^1^H NMR peak assignable to a hydrogen bonded proton seems to indicate a similar situation, i.e., dimeric IbuH confined in CDNS linked by IbuH∙∙∙IbuH interactions. However, it should be kept in mind that other types of hydrogen bonds may be established, such as those between IbuH and the free OH groups of CD or the COOH and amino functional groups of the cross-linker. The acidic IbuH form present in the network can be motivated by considering that, even if the swelling of CDNS is achieved by adding an aqueous basic solution of sodium carbonate [9], the final pH of the hydrogel is slightly acidic. Moreover, water removal by lyophilisation induces an increased protonation of IbuH, due to the concentration of the system.

#### ^13^C CP-MAS NMR spectroscopy

In order to get more information on the CDNS polymer structure, polymer–drug interactions and drug mobility, we recorded the ^13^C CP-MAS spectra for several samples of free CDNS and the corresponding drug-loaded samples. Three different types of samples were studied: 1) free polymers CDNS(1:4) and CDNS(1:8); 2) pure IbuNa; 3) CDNS(1:4)-IbuNa and CDNS(1:8)-IbuNa.

**Figure 4:**
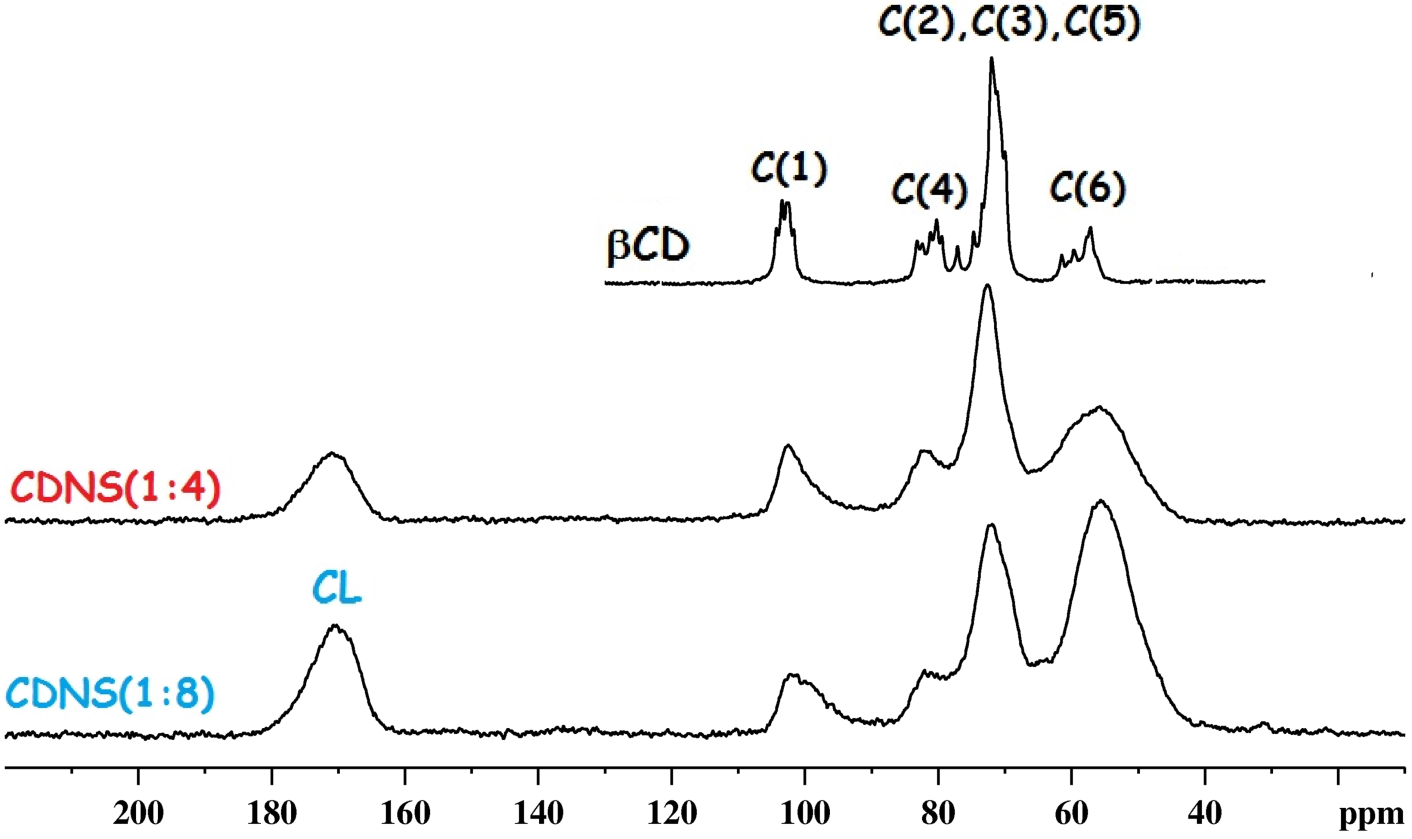
^13^C CP-MAS NMR spectra for samples of β-CD (top), CDNS(1:4) (middle) and CDNS(1:8) polymers (bottom). CL indicates the carbonyl group of the cross-linking agent.

Both spectra of the CDNS polymers, reported in Figure 4, show broad lines characteristic of amorphous materials. The top trace of Figure 4 shows the characteristic spectrum of crystalline β-CD, along with the peak assignment already known from the literature. The peak assignment is a guide for the interpretation of the spectra of CDNS. The observed chemical shifts follow the order C(1), C(4), [C(5), C(3) and C(2)] and C(6), in increasing order of shielding. For both CDNS polymers, the peak assignment is reported in Table 1.

**Figure 5:**
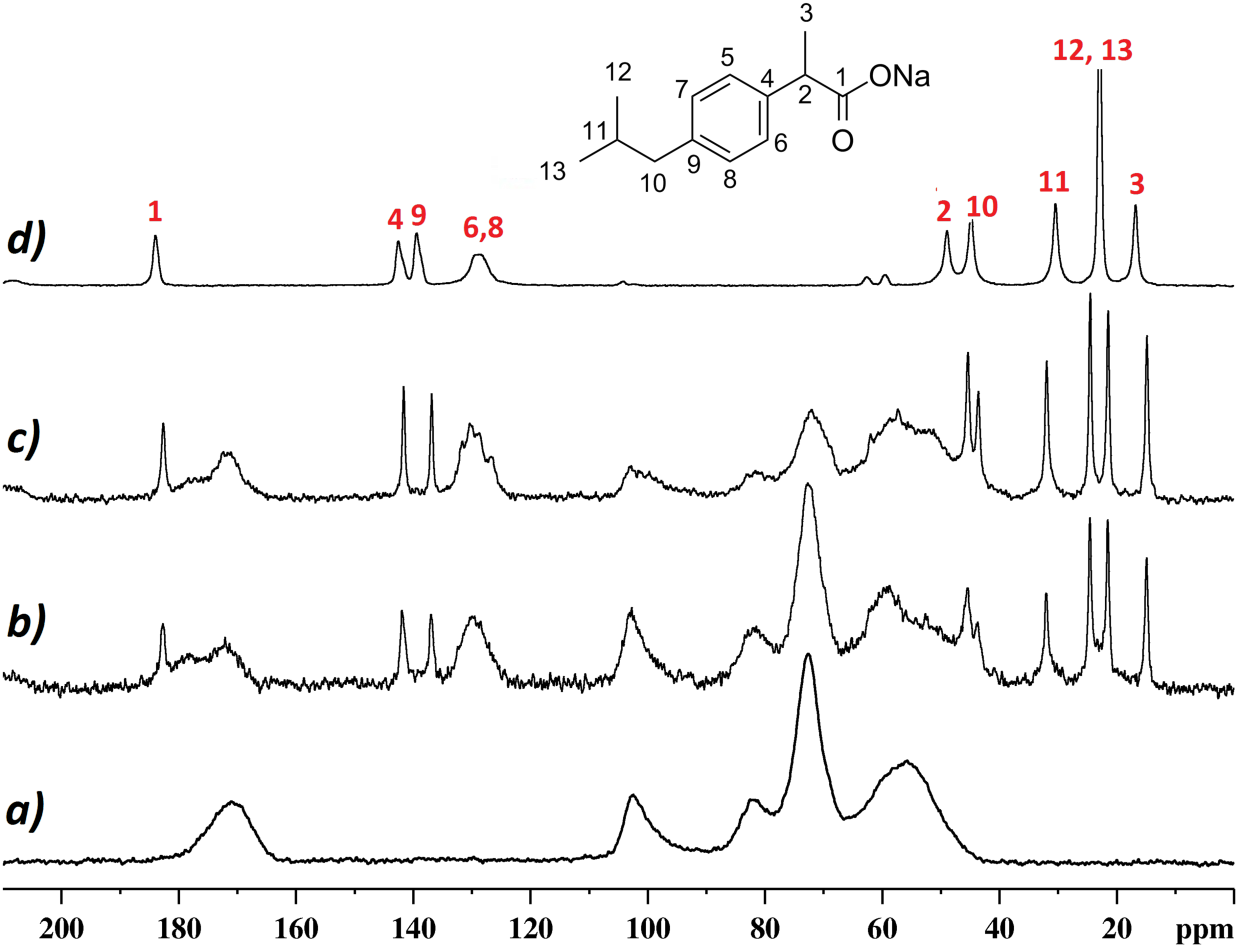
^13^C CP-MAS NMR spectrum of: a) CDNS(1:4) polymer; b) CDNS(1:4)-IbuNa system; c) CDNS(1:8)-IbuNa system; d) pure IbuNa (reference spectrum). The molecular structure and atom numbering of IbuNa are shown in the inset. The notation CDNS-IbuNa indicates the original preparation, irrespective of the conversion of the sodium salt into the undissociated acid.

The ^13^C CP-MAS spectrum of IbuNa is reported in Figure 5 (top trace). It was acquired as reference spectrum of the drug molecule and the resonances assigned according to Geppi et al. [24]. The spectrum shows sharp lines (line-width range: 80–100 Hz) specific of a crystalline sample. Ibuprofen is a small molecule characterized by an interesting internal dynamic behaviour due to two main molecular fragments: the alkyl chain and the aromatic ring. The internal motion was studied in detail using solid-state ^13^C NMR spectroscopy at different temperatures by Carignani et al. [25]. The authors identified two types of motion with a different time scale: 1) the rotations of the two methyl groups of the isobutyl moiety, occurring in the fast regime, 2) the π-flip of the phenyl ring belonging to the intermediate motional regime.

Both rotational motions are fast enough to observe an average isochronous signal for the methyl carbon atoms (12 and 13) as well as for the aromatic protonated carbons (5,6–7,8). It is worth reminding that a different mobility was observed for ibuprofen in its acid form (IbuH) which shows hindered rotational motion for both molecular fragments.

The ^13^C CP-MAS NMR spectra of the CDNS samples without and with ibuprofen are also shown in Figure 5. A comparison of the reported spectra does not highlight any observable change of the linewidth and chemical shift of the CDNS signals after loading the polymers with IbuNa. This is a clear indication that adding the drug to the CDNS does not influence significantly the nanosponge porous structure. From the point of view of the encapsulated drug, the ^13^C CP-MAS spectra show that the peaks due to ibuprofen retain high resolution even when the drug is encapsulated in the nanosponges, thus proving that the drug is in a crystalline form inside the rigid polymeric network. However, two important findings should be highlighted here. The peaks assigned to C12 and C13 of IbuNa – which are isochronous in the reference sample (Figure 5d) and they give rise to a singlet at ca. 23 ppm – provide two separate signals when IbuNa is loaded onto the polymers, as clearly visible in Figure 5b and c. This finding further supports what described in the previous section: the guest molecule ibuprofen, originally added to the polymeric matrix as the sodium salt IbuNa, is present, in the CDNS(1:4) and CDNS(1:8) nanosponges, as the corresponding acid form IbuH. Indeed, the chemical shift of ibuprofen signals from the spectra of Figure 5b and 5c match with those reported in the literature for the ^13^C CP-MAS NMR spectrum of crystalline IbuH [25]. The indication that IbuH is not significantly interacting with the polymeric backbone and that the ^13^C CP-MAS NMR chemical shift of encapsulated IbuH match those of pure, crystalline IbuH provide useful, additional information to spot on the nature of the hydrogen bond network introduced in the previous section. As significant IbuH···CDNS interactions are not detected, the hydrogen bond detected via ^1^H NMR and discussed in the previous section is likely to involve two IbuH units in the formation of dimers.

#### Dynamics of cross polarization

**Figure 6:**
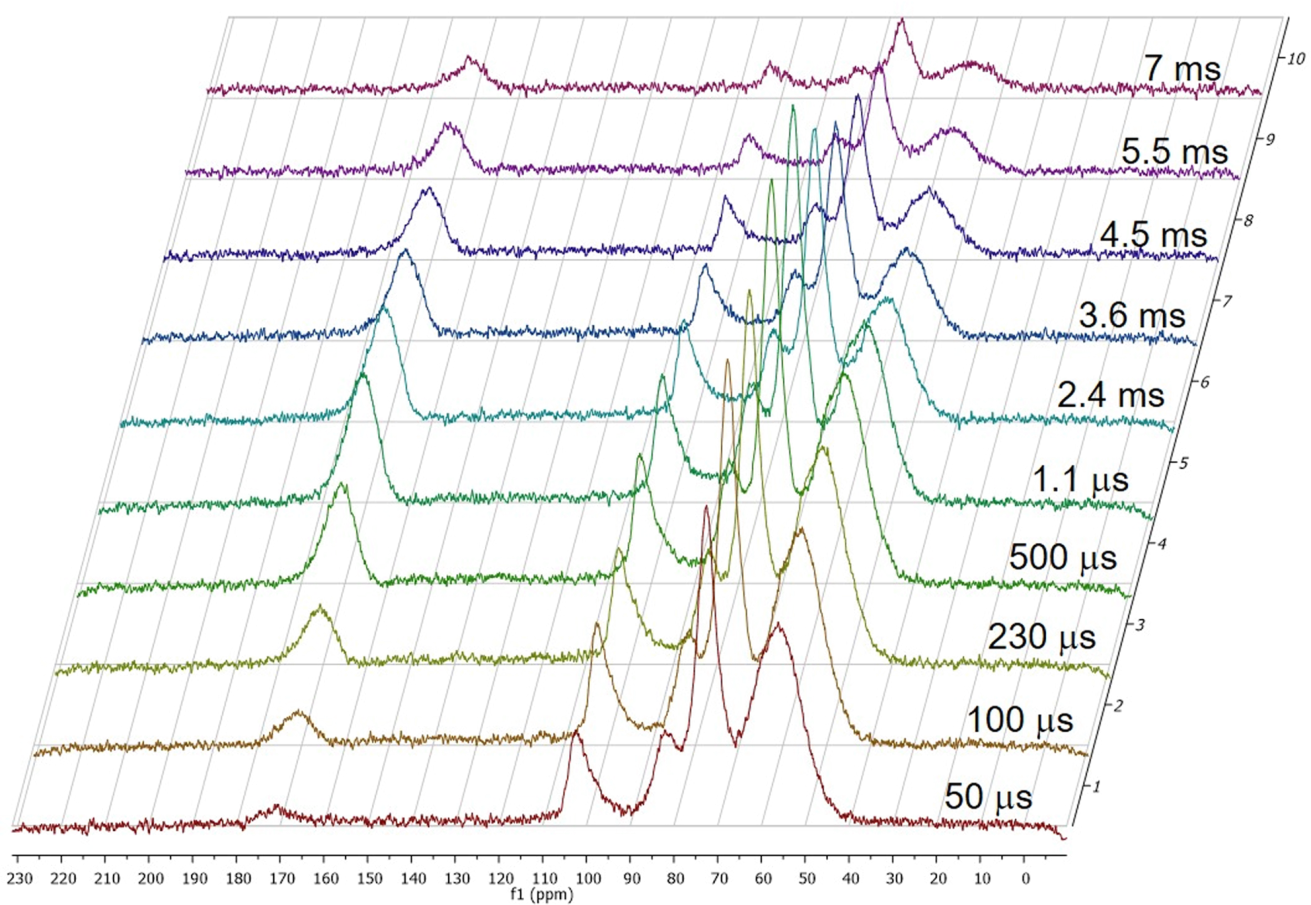
^13^C CP-MAS NMR spectra of CDNS(1:4) acquired with CP variable contact time in the range 50 μs–7 ms.

**Figure 7:**
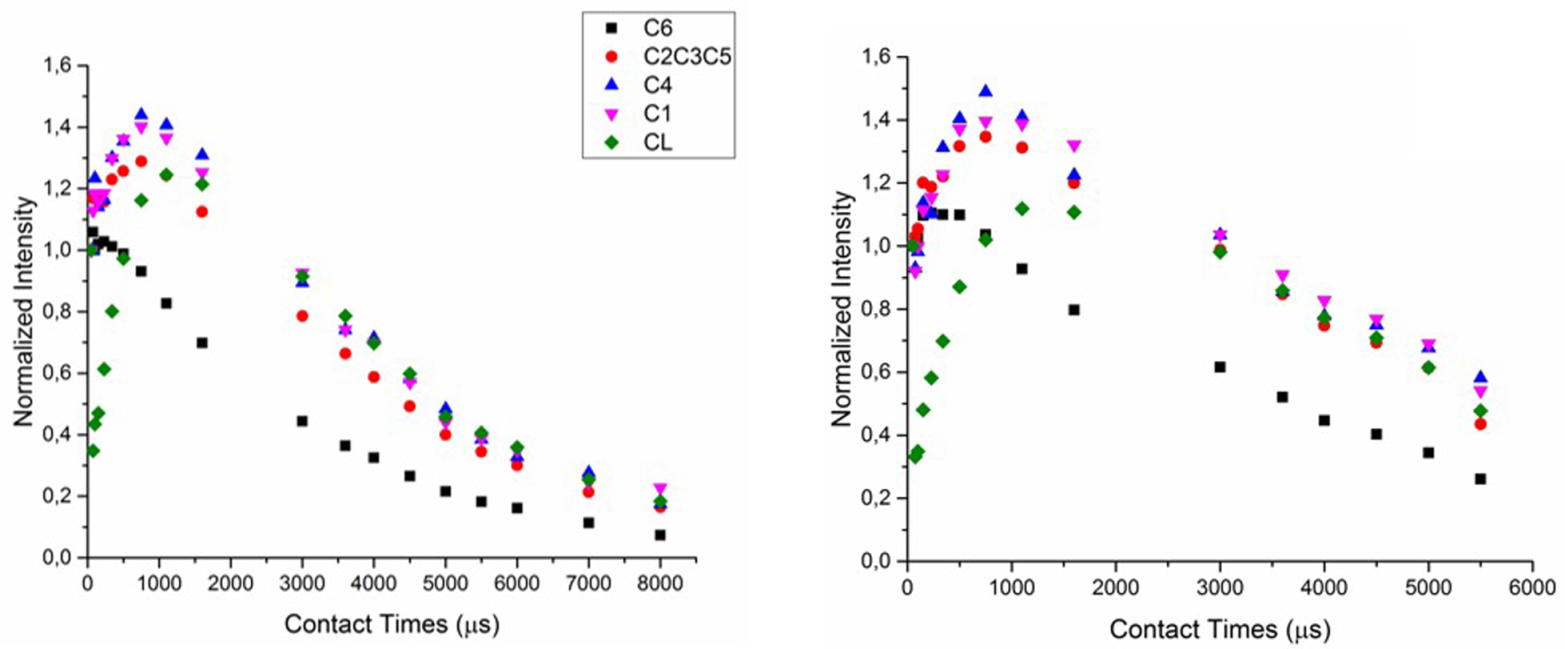
Time dependence of ^13^C magnetization for CDNS(1:4) (left) and CDNS(1:8) (right) polymers.

We applied variable contact time (VCT) ^1^H-^13^C CP-MAS NMR techniques to study the CP kinetics of both the CDNS polymers. As an example, the array of ^13^C CP-MAS spectra acquired at the MAS rate of 10 kHz and with increasing contact time for cross-polarization is shown in Figure 6 in the case of CDNS(1:4). We can observe that at 100 μs all the carbon atoms are polarized, i.e., the signal intensity is high enough to generate an observable ^13^C CP-MAS NMR spectrum. The maximum signal intensity *S*_CPMAX_ is observed around 750 μs for all the cyclodextrin carbon atoms and around 1100 μs for the carbonyl groups of the cross-linker. Such small values indicate a fast cross-polarization process, consistent with a rigid polymeric system. Therefore, strong heteronuclear dipolar interactions are present and capable to facilitate an effective magnetization transfer from the proton reservoir to various carbon atoms. The CP kinetics in both CDNS systems follow the classical *I–S* model. The signal intensity *I*(t), expressed by the peak area, versus the CP contact time *t* are reported in Figure 7 for all the carbon atoms.

The experimental curves can be fitted by using Equation 2, thus providing the values of the proton relaxation parameter *T*_1ρ_. The results are collected in Table 1 for CDNS(1:4), CDNS(1:8). For clarity, also the literature *T*_1ρ_ values related to monomeric β-cyclodextrin are listed [26]. All the carbon atoms revealed short *T*_1ρ_ values in the range of 3–5 ms and similar for β-CD C atoms and the carbonyl atom of the cross-linker, suggesting that their chemical environments are equally proximate to the ^1^H reservoirs. This result is indicative of the homogeneous nature of the polymer sample. The only exception to this general trend is observed for the cross-linker C atom of CDNS(1:8). In such a case, *T*_1ρ_ of 3 ms was detected, while all the other C atoms show the constant value of 5 ms. This finding confirms a certain degree of heterogeneity in CDNS(1:8) connected to the carbonyl groups of the CL in this polymer, in agreement with the observation previously reported for the same system in the gel state [9].

**Table 1:** Chemical shift (ppm), and proton relaxation *T*_1_ρ (ms) of the carbon atoms of CDNS(1:4), CDNS(1:8) polymers and pristine β-CD.

Carbon atoms	Chemical shift^a^	*T*_1_ρ CDNS(1:4)^a^	*T*_1_ρ CDNS(1:8)^a^	*T*_1_ρ β-CD^b^

CL (carbonyl)	170	3.3	3.0	
C(1)	102	3.3	5.0	2.6
C(4)	82	3.3	5.0	2.4–2.6
C(2), C(3), C(5)	72	3.3	5.0	2.2–2.5
C(6) + CL (methylene)	56	3.0	5.0	2.7

^a^This work. ^b^From ref. [26].

**Figure 8:**
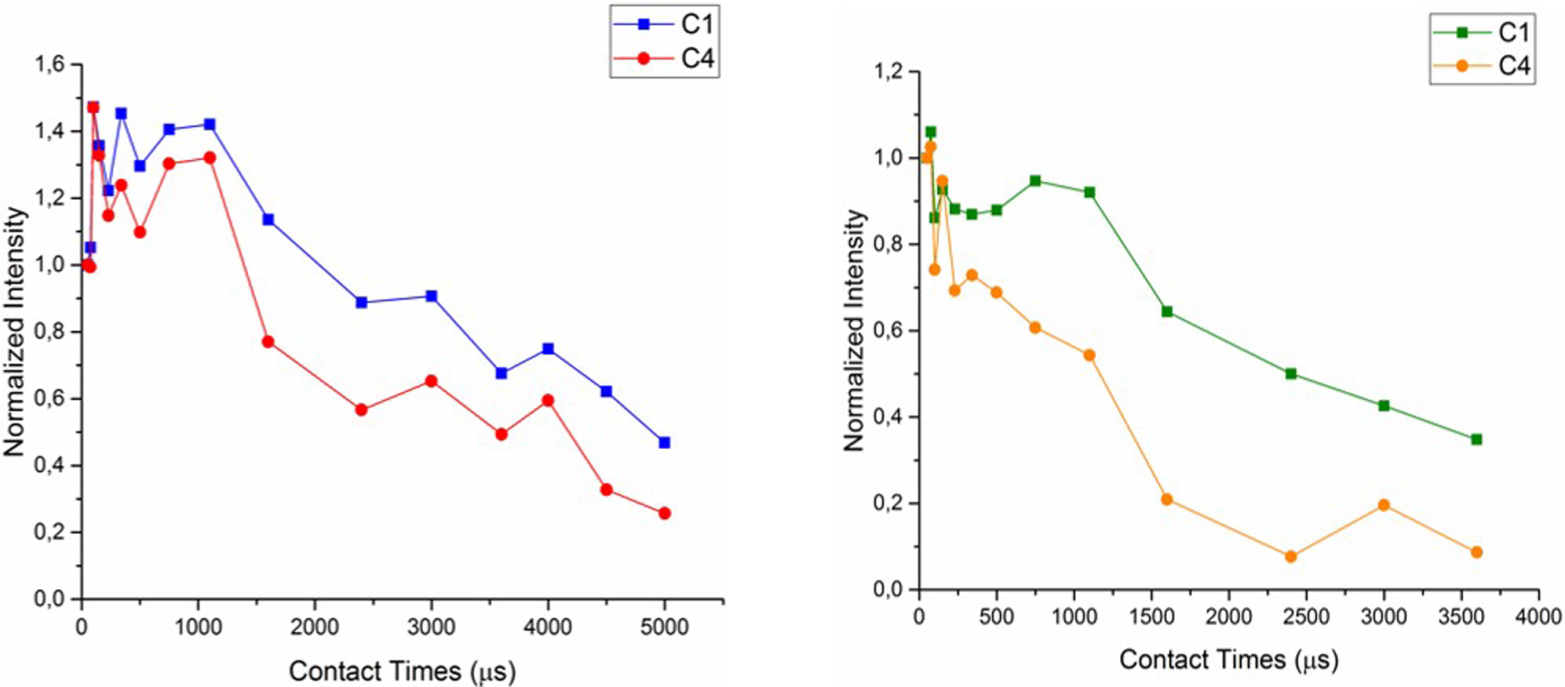
^1^H-^13^C CP oscillatory kinetics for the carbon C(1) and C(4) of CDNS(1:4)-IbuNa sample *(*left*)* and CDNS(1:8)-IbuNa (right). The notation CDNS-IbuNa indicates the original preparation, irrespective of the conversion of the sodium salt into the undissociated acid.

**Figure 9:**
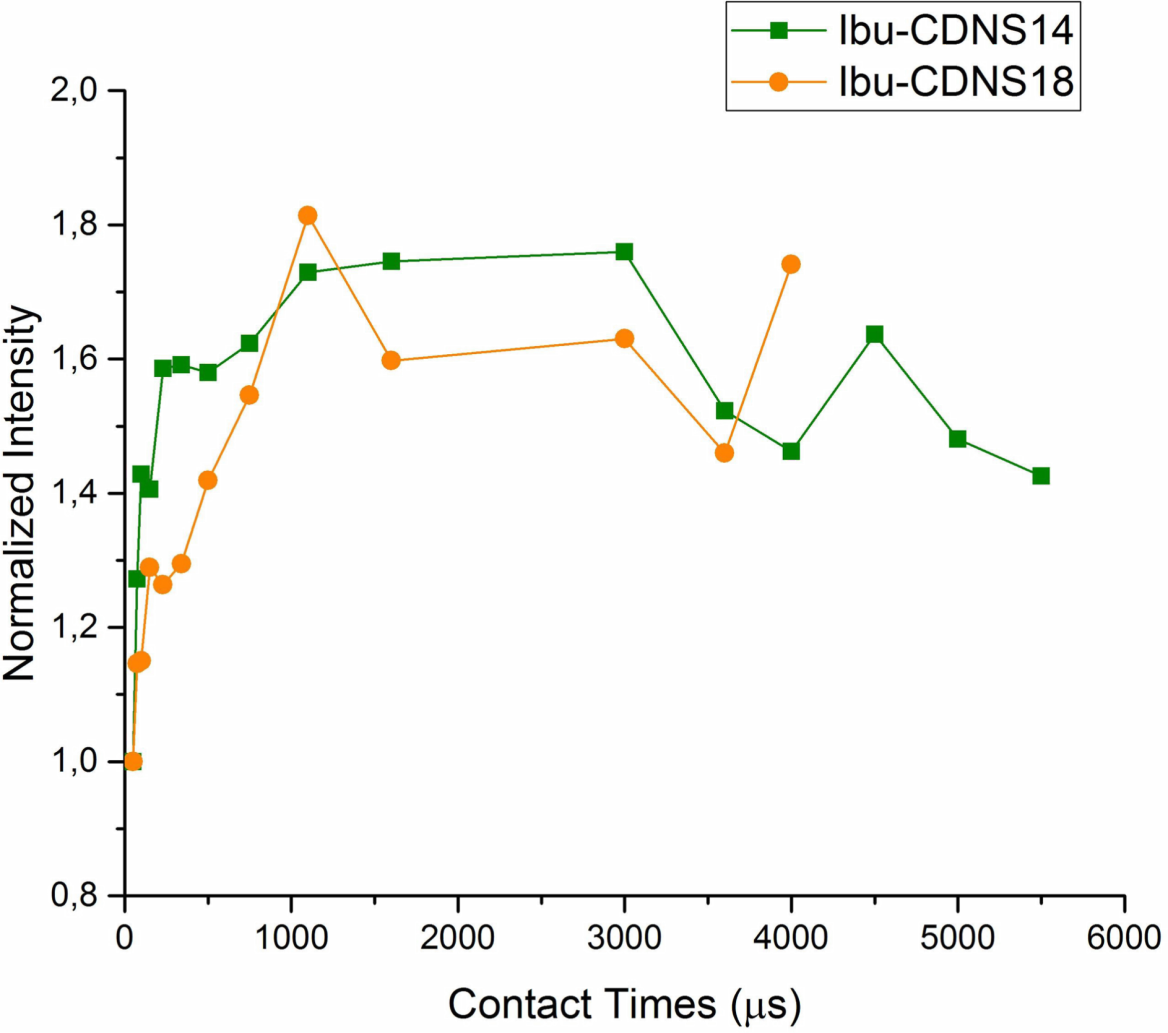
^1^H-^13^C CP oscillatory kinetics for the ibuprofen aromatic carbon atoms C(6,8) in samples CDNS(1:4)-IbuNa and CDNS(1:8)-IbuNa. The notation CDNS-IbuNa indicates the original preparation, irrespective of the conversion of the sodium salt into the undissociated acid.

A different, more complex, kinetic behaviour is obtained for both the CDNS-IbuNa samples. For all the carbon atoms of the CDNS polymer as well as for all the carbons of ibuprofen, our VCT results followed a non-classical kinetic model (I–I*–S model) corresponding to oscillatory polarization transfer. The results for the C(1), C(4) atoms of the CDNS(1:4) and CDNS(1:8) polymers are shown in Figure 8 and Figure 9, respectively. The dipolar oscillations modulate the magnetization build-up curve at short mixing times up to 750 μs, then the time constants (proton spin diffusion *T*_diff_ and proton spin–lattice relaxation in the rotating frame *T*_1_ρ) determine the magnetization decay.

The CP kinetic curve of ibuprofen (aromatic carbons 6, 8) is shown in Figure 9. In this case, the magnetization intensity rises at short mixing times (in the range 50–1100 μs) and then approaches a plateau which means that *T*_1ρ_ (H) is infinitely long.

The process of drug loading in both CDNS polymers influences the dynamic behaviour of both the polymer and the drug due to strong heteronuclear dipolar interactions rising in a rigidly connected system. A more detailed analysis of these systems would need a deeper theoretical data treatment. Additionally, in order to achieve an accurate fitting, some relaxation parameters need to be determined with different solid state experiments such as depolarization or the TORQUE [27] experimental method, which is beyond the aim of this work. In the economy of the present work, the most important conclusion of this section is that the VCT profiles of the free polymers – CDNS(1:4) and CDNS(1:8) – and the corresponding drug-loaded formulations – CDNS(1:4)-IbuNa and CDNS(1:8)-IbuNa – fit two different dynamic models: the commonly observed *I–S* scheme and the non-classic, less common I–I*–S model, respectively. The changes observed in the dynamics of the system after the drug loading point out that the polymer and the drug are in contact (dipolar contact) and that the magnetization is transferred from the protons (of both polymer and drug) to the CDNS carbons. This allows to conclude that a supramolecular architecture is formed, in spite of the fact that the initial structure of the CDNS polymer is retained.

### Powder X-ray diffraction

**Figure 10:**
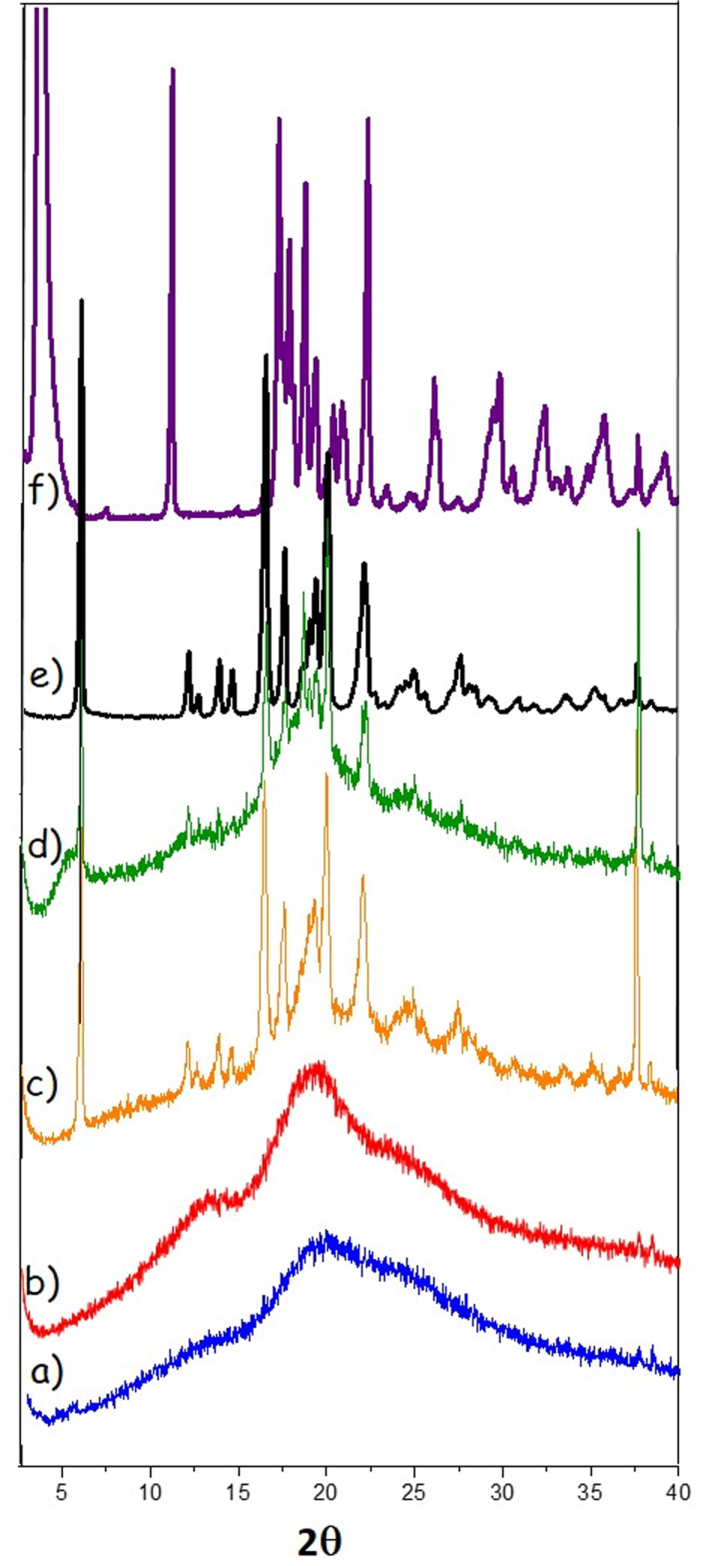
PXRD patterns of: a) CDNS(1:8), b) CDNS(1:4), c) CDNS(1:8)-IbuNa loaded, d) CDNS(1:4)-IbuNa loaded, e) IbuH, analytical standard, f) IbuNa, analytical standard, The notation CDNS-IbuNa indicates the original preparation, irrespective of the conversion of the sodium salt into the undissociated acid.

The diffraction patterns of both CDNS used in the present work (Figure 10a and b) show a totally amorphous structure in agreement with the results of ^13^C CP-MAS NMR. Once loaded with ibuprofen sodium salt, the PXRD profiles of both CDNS (Figure 10c and d) show the amorphous structure of CDNS along with peaks belonging to ibuprofen. The ibuprofen Bragg peaks are intense and sharp, typical for a high ordered crystalline structure. Their positions are in agreement with the diffraction pattern of the acidic racemic form of ibuprofen, thus confirming that, after the lyophilisation, the IbuNa originally added to the nanosponge is turned into the undissociated acid IbuH. For comparison, the spectrum of a standard sample of pure, racemic ibuprofen in its acidic form (IbuH) is also reported in the figure (Figure 10e). The peak positions match perfectly the literature reference [28] and they are clearly distinguishable from the reflexes of IbuNa, shown in upper traces of the figure (Figure 10f).

As a final remark, the PXRD patterns show that ibuprofen entrapped into the nanosponge network still retains crystallinity, thus confirming that the formation of the polymer-drug association is a mild process.

## Conclusion

The mesoporous materials CDNS(1:4), CDNS(1:8) in their free form and loaded with ibuprofen sodium salt were prepared and thoroughly characterized using solid-state ^1^H Fast MAS and ^13^C CP-MAS NMR spectroscopy. The main conclusions can be summarized as follows:

1. Ibuprofen sodium salt is converted into its acid form IbuH after the lyophilisation of the hydrogels obtained by swelling CDNS(1:4) and CDNS(1:8) with aqueous IbuNa solutions.

2. ^1^H Fast MAS NMR spectra reveal that ibuprofen loaded in the CDNS polymeric scaffold does not form H-bonds with the polymer, but rather it crystallizes as a dimer.

3. ^13^C CP-MAS spectra allow the characterization of the free CDNS polymers as amorphous materials. ^13^C CP-VCT experiments show that the investigated cyclodextrin nanosponges are rigid and homogeneous.

4. ^13^C CP-MAS spectra of the CDNS and the CDNS-drug systems do not show observable chemical shift changes, thus ruling out significant structural changes (e.g., conformational or structural) for the polymer backbone after the entrapment of the drug.

5. Conversely, ^13^C CP-VCT NMR experiments provide evidence that the kinetic behaviour of all the carbon atoms of the CDNS polymers dramatically changes upon drug loading, thus providing a dynamic fingerprint of the formation of a supramolecular architecture. The latter is characterized by the formation of IbuH domanins in the pores of the 3D structure of the polymeric network. The experimental data show that the inclusion of IbuH in the CD cavity – namely the formation of a host–guest inclusion complex – does not occur, confirming the situation already described by HR-MAS NMR data for the same system in the gel state [9] and outlining a different scenario on passing from monomeric CD, capable to form well characterized inclusion complexes with IbuH [29–31] to CDNS of the present work. A possible explanation relies upon the availability of the CD cavity within the polymeric network. The importance of this factor is particularly clear in the case of polymers obtained by monomers containing cyclodextrin units as dangling groups, e.g., glycidylmethacrylate-mono-6-amino-6-deoxy-β-CD (GMA-NH_2_-β-CD) co-polymerized with ethylene dimethacrylate [32]. The corresponding cross-linked polymers are thus decorated with cyclodextrin units, easily accessible to small molecules to give rise to inclusion complexes. This type of polymers showed excellent efficiency as chiral selectors for the enantioseparation of racemic ibuprofen [32]. In our case, the primary OH groups of CD units react with EDTA dianhydride to form a cross-linked polyester. As a matter of fact, this type of polycondensation makes the CD cavity less available compared to the former case. The overall result is that the guest molecules are mainly confined in the pores formed by the polycondensation process rather than forming a genuine inclusion complex with the single CD units. From a practical point of view, our class of polymerized cyclodextrins can be conveniently exploited as sorbent or scaffold by exploiting the pores of the polymer network to encapsulate the guest molecules. However, it should be stressed that CD nanosponges similar to ours and prepared by using citric acid as cross-linker, showed a different behaviour towards ibuprofen: the authors provided evidence of inclusion complex formation as the main mechanism of absorption [33]. We may conclude that the type of monomer and the cross-linker agent both play a key role in driving the absorption towards inclusion complexes or segregation in the polymer pores, although a sufficient number of data is still missing to formulate a general model.

As a final remark, the possibility of solid-state reactions involving the active components should be taken into account when designing scaffolds for in situ release. The methodologies here described can be conveniently included in the tool-case to monitor the molecular state and dynamics of the host–guest systems.

## Experimental

### Materials and measurements

The detailed procedures for the synthesis of CDNS and the preparation of the corresponding hydrogels containing IbuNa can be found and seen in [34]. In the following, we report the essential information on the synthesis and the experimental details for the NMR and XRD experiments.

#### Nanosponges preparation and drug loading procedure

The synthesis of CDNS(1:4) and CDNS(1:8) was done according to the protocol previously described [9]. The drug loading procedure consists of three fundamental steps:

1. A stock solution (0.27 M) of ibuprofen sodium salt was prepared by dissolving 308 mg of IbuNa together with 68 mg of Na_2_CO_3_ in 5 mL of water.
2. 3 mL of the solution (1) were then added to a weighted amount (400 mg) of CDNS polymer in both preparations.
3. Homogeneous hydrogels were then obtained in 1 h. The measured pH of the gels was 6.5. The samples are then freeze-dried overnight.

#### Solid state NMR spectroscopy

^1^H NMR spectra were recorded on VNMRS spectrometer operating at 600 MHz (51 mm bore Oxford superconducting magnet) equipped with a 1.6 mm Triple Resonance Fast MAS probehead. Sample rotation frequencies were 20–40 kHz for ^1^H Fast MAS NMR experiment.

^13^C CP-MAS NMR spectra were collected at 125.77 MHz on the 500 MHz NMR Spectrometer AvanceTM 500 operating at a static field of 11.7 Tesla (superconducting ultrashield magnet) and equipped with a 4 mm MAS probe. All the samples were prepared by packing the powder in Zirconia rotors (ZrO_2_), closed with Kel-F caps (80 μL internal volume); the spinning speed (MAS) was optimized at 10 kHz.

Cross-polarization (CP) spectra, under Hartmann–Hahn conditions, were recorded with a variable spin-lock sequence (ramp CP-MAS), a relaxation delay d1 = 4 s, and a ^1^H π/2 pulse-width of 4.2 μs was employed. The contact time (CT) for the VCT experiments was varied in the range of 50 μs to 7.0 ms; 1200 scans for each experiment were acquired.

The CP-MAS signals were approximated by Lorentzian function curve fitting analysis. The baseline and the deconvolution of the peaks were performed with OriginPro 2016 software. The accordance between experimental and deconvoluted curves is expressed as R^2^ value which is >0.98 for all of the analyzed spectra.

#### X-ray diffraction experiments

The powder X-ray diffraction (PXRD) experiments were performed with a Bruker D2 Phaser X-ray powder diffractometer using CuKα radiation. The data were collected in the 2θ range 2.3–40° with a step size of 0.02° and a counting time of 0.4 s per step, a primary slit module of 0.6 mm, air scatter screen module 1 mm and secondary slit module 8 mm.
